# Single cell RNA sequencing reveals regional heterogeneity of hepatobiliary innate lymphoid cells in a tissue-enriched fashion

**DOI:** 10.1371/journal.pone.0215481

**Published:** 2019-04-25

**Authors:** Anna L. Peters, Zhenhua Luo, Jun Li, Reena Mourya, Yunguan Wang, Phillip Dexheimer, Pranav Shivakumar, Bruce Aronow, Jorge A. Bezerra

**Affiliations:** 1 Department of Pediatrics, Division of Gastroenterology, Hepatology and Nutrition, Cincinnati Children’s Hospital Medical Center, Cincinnati, Ohio, United States of America; 2 Department of Pediatrics, University of Cincinnati College of Medicine, Cincinnati, Ohio, United States of America; 3 Department of Pediatrics, Division of Bioinformatics, Cincinnati Children’s Hospital Medical Center, Cincinnati, Ohio, United States of America; Texas A&M University, UNITED STATES

## Abstract

IL-33 promotes type 2 immunity, epithelial repair, and tissue fibrosis by activating group 2 innate lymphoid cells (ILC2). ILC2 lack all known surface markers of mature T, B, NK, and myeloid cell lineages (Lin^neg^), express the IL-33 receptor ST2, and release type 2 cytokines which contribute to cholangiocyte proliferation and activation of hepatic stellate cells. This pathway results in massive proliferation of the extrahepatic bile duct (EHBD) but also exacerbates liver fibrosis, suggesting that there may be tissue-specific subpopulations of IL-33-induced ILC. To determine the tissue-specific subsets of ILC in the hepatobiliary system, we analyzed CD45^+^Lin^neg^ mononuclear cells from IL-33 treated adult Balb/c mouse liver or EHBD by single cell RNA sequencing. Principal component analysis identified 6 major CD45^+^Lin^neg^ cell classes, two of which were restricted to the EHBD. One of these classes, biliary immature myeloid (BIM) cells, was predicted to interact with ILC2 by a network of shared receptor-ligand pairs. BIM highly expressed Gp49 and ST2 receptors on the cell surface while lacking surface expression of markers for mature myeloid cells. In conclusion, single cell RNA sequencing identified IL-33 responsive cell groups regionally confined to the liver or extrahepatic bile duct, including a novel population of CD45^+^Lin^neg^ Gp49-expressing mononuclear cells.

## Introduction

Innate lymphoid cells (ILC) are distributed at epithelial sites early in life to uniquely respond to tissue injury and initiate and participate in immune responses. ILC express CD45, IL-7Rα and other immune activation markers but lack all known lineage markers (Lin^neg^) for T, B, myeloid, and NK cells [1–3]. Among ILCs, the group 2 innate lymphoid cells (ILC2) respond to IL-33, a member of the IL-1 family of cytokines released upon epithelial damage to promote type 2 immunity to parasites, epithelial repair, and tissue fibrosis in both mice and humans in various tissues including skin, lung, GI tract, liver and bile duct [4,5]. ILC2s release IL-13 and other type 2 cytokines, which clear parasitic infections but play pathogenic roles in exacerbating asthma and allergic immune responses [6].

Within the hepatobiliary system, we and others have shown that IL-33 activates hepatic ILC2 to produce IL-13, which induces massive proliferative expansion of the epithelium and peribiliary glands (PBG) of the extrahepatic bile duct (EHBD). This molecular circuit is protective in a mouse model of biliary atresia, as evidenced by the fact that 1) a subset of patients with biliary atresia overexpress IL-33, 2) blockade of IL-33 signaling in a mouse model of biliary atresia induced by Rhesus rotavirus (RRV) infection exacerbates disease, and 3) administration of IL-33 to RRV-infected mice is protective against EHBD obstruction [7]. In humans biliary atresia leads to rapidly progressive biliary cirrhosis, often requiring liver transplantation for long-term survival [8]. Experimentally, IL-33 also promotes the development of cholangiocarcinoma in genetically predisposed mice [7,9]. In this context, previous reports have shown that the IL-33/ILC2/IL-13 axis exacerbates liver fibrosis in mice, with circumstantial evidence of the activation of this axis in human cirrhosis [10–12]. These different results suggest that the response of hepatobiliary ILC to IL-33 is tissue-specific; however, the spectrum of ILC subclasses induced by IL-33 within the hepatobiliary system is unknown. In this study, we investigated the heterogeneity of tissue-specific subclasses of hepatobiliary ILCs by performing single cell RNA sequencing on CD45^+^Lin^neg^ mononuclear cells isolated from the liver or EHBD after IL-33 administration. Using a gene-group guide technique, we identified 6 major classes, one of which was restricted to the EHBD and expressed high levels of *Gp49*, *IL1rn*, and *Csf2r* consistent with immature myeloid cells.

## Materials & methods

### Antibodies and reagents

Recombinant mouse IL-33 was from Biolegend. BrdU was purchased from BD Biosciences. DMEM was from Gibco, and collagenase D from Sigma.

The following primary antibodies were used for flow cytometry: anti-mouse CD16/32 (2.4G2), PerCP Cy5.5 anti-TER119 (Ter119), PerCP-Cy5.5 Gr-1 (RB6-8C5), PerCP Cy5.5 anti-CD8 (53–6.7), PE-Cy7 anti-ckit (2B8) from eBiosciences. The following antibodies used for flow cytometry were from Biolegend: FITC anti-F4/80 (BM8), PE-Cy7 anti-ICOS (C398.4A), PE or AF647 anti-Gp49 (H1.1), PE or AF700 anti-CD45 (30-F11), APC anti-CD127 (A7R34), APC-Cy7 anti-Ly6C (HK1.4), AF700 anti-Ly6G (1A8), Pacific Blue anti-CD44 (1M7), Pacific Blue anti-CD11b (M1/70), PerCP Cy5.5-conjugated anti-F4/80 (BM8), anti-B220 (RA3-6B2), anti-CD3 (145-2C11), anti-CD4 (RM4-5), anti-CD11b (M1/70), anti-CD11c (N418), CD49b (DX5), and FcεRIα (MAR-1). Lineage cocktail included PerCP Cy5.5-conjugated anti-CD3, anti-CD4, anti-CD8, anti-B220, anti-CD11b, anti-CD11c, anti-CD49b, Gr-1, F4/80, Ter119 and FcεRIα, which were also from BioLegend.

The following antibodies and reagents were used for immunofluorescence: biotin anti-BrdU (Abcam), rabbit anti-cytokeratin antibody (Dako), DyLight 488–conjugated donkey anti-rabbit and DyLight 594–conjugated streptavidin antibodies (Jackson Immunoresearch), ProLong Gold antifade reagent with DAPI for nuclear staining (Invitrogen), Target Retrieval Solution and Antibody Diluent (both from Dako).

### Mouse lines and experimental challenges

Adult (6–12 week) age- and sex-matched Balb/c mice were purchased from Charles River Laboratories or bred in house in a specific pathogen free facility. To induce bile duct proliferation and expansion of Lin^neg^ lymphocytes, 1μg IL-33 or PBS was injected intraperitoneally (i.p.) into adult Balb/c mice for 1 or 4 consecutive days. For in vivo bile duct proliferation assays, mice were treated with BrdU 1mg i.p. 2 hours prior to sacrifice. Mice were euthanized via CO_2_ inhalation.

### Single cell RNA sequencing and computing cell-cell interactions

PBS or 1μg rIL-33 was administered daily to Balb/c mice i.p., and mice were sacrificed after 1 day (1d) or 4 consecutive days (4d) of treatment. Liver mononuclear cells were isolated from 8 mice per treatment group (PBS, 1d IL-33, 4d IL-33); EHBD mononuclear cells were isolated and pooled from 16 mice after daily administration of rIL-33 for 4d. Because the number of CD45^+^ mononuclear cells existing in the PBS-treated EHBD was exceedingly low, and would require an excessive number of mice in order to obtain sufficient cells to analyze, we did not include this control group. Each liver or up to 4 bile ducts were minced into 5ml serum-free DMEM + 1mg/ml collagenase D and incubated at 37°C for 30min. Mononuclear cells were isolated via Percoll gradient as described [7]. Mononuclear cells were stained with fluorochrome-conjugated antibodies to Lineage markers and CD45. CD45^+^Lin^neg^ cells were sorted to >99% purity using a FACSAria II cell sorter (BD Biosciences), resuspended at a concentration of 30,000–40,000 cells/μl and loaded onto a C1 5–10μm chip for single cell RNA preparation on the Fluidigm platform.

Transcript expression was quantified using kallisto v0.42.2.1 with the “—bias” parameter. Transcript definitions were retrieved from the UCSC mm10 knownGenes tables in November 2014, and gene level expression was calculated as the sum of expression (in TPM) of each constituent isoform.

Unsupervised cell clustering was carried out by a re-iterative cluster analysis pipeline constructed in Python, which works iteratively in the following 3 steps: dimension reduction (feature selection), clustering, and signature gene identification. Dimension reduction was performed at the first clustering iteration by principle component analysis (PCA) using the Scikit-learn package in Python (n_component = 0.8). In the following iterations, a pooled list of genes (training set) that are highly expressed in at least one cell cluster were used as input features (described below). Clustering of cells in the dimension-reduced expression data was carried out using the hierarchical clustering algorithms with average linkage on Pearson correlation space. Optionally, a second round of clustering was applied on single cells in a major cluster to divide them further into subclusters. Following cell clustering, genes highly expressed in each clustered cell population were identified through integrating up to 4 gene rankings using the Rank Product algorithm[13]. These rankings derived from the Wilcoxon rank-sum test and expression fold change (FC) comparing the target cell cluster or subcluster with other clusters or subclusters, respectively. Top ranked genes from each cell population were then pooled and used as input training set for the next clustering iteration [14].

To assign a cell identity to the cell classes identified by scRNA-SEQ, the top 200 most highly expressed genes were imported into ToppGene (toppgene.cchmc.org). Additionally, the gene list was manually curated to enrich for transcription factors, cytokines, and receptor/ligand pairs. To determine potential cell-cell interactions, the original top 200 gene set for each cell class was further refined to include only those genes which expressed in >50% of the cells in each class at a log_2_(TPM+1) value > 2 and not expressed in any other cell classes. These were termed “unique genes.” Unique genes in each cell class present in the EHBD were then used to determine potential receptor-ligand interactions. Receptor-ligand information was compiled from the DIP database [15,16]. Unique genes in each cell class were then imported into the Circlize version 0.4.1 in R (version 3.1.1) to generate a circle interaction diagram.

### RNA sequencing and qRT-PCR

RNA isolation from liver and bile duct tissue after 1 or 4d of IL-33 treatment was performed as described previously. Paired-end RNA sequencing was performed in triplicate in each experimental group as described previously [17] with at least 90 million reads per sample. For qRT-PCR experiments, cDNA synthesis, and quantitative PCR amplification using the Brilliant III SYBR Green QPCR Master Mix gene expression assay kit and the Mx3005P system (Stratagene, Agilent Technologies) were performed as described previously [17].

### Flow cytometry and cell sorting

According to previously published protocols [7], cells were re-suspended in PBS supplemented with 5% fetal calf serum and incubated at 4°C with combinations of fluorochrome-conjugated antibodies. Cells were analyzed on a FACSCanto flow cytometer (BD Biosciences) or were purified with a FACSAria II cell sorter (BD Biosciences). Data were analyzed with FlowJo software, version 9.7.7.

### Quantification of cholangiocyte proliferation by BrdU uptake

Extrahepatic bile ducts and liver sections were dissected under a microscope, fixed in 10% formalin, and embedded in paraffin, and sectioned longitudinally. After deparaffinization, antigen retrieval was performed with DAKO antigen retrieval solution. 5μm sections were stained with fluorescent antibodies to cytokeratin (CK) and BrdU, and slides coverslipped with ProLong Gold with DAPI antifade reagent and examined by fluorescence microscopy as described [7]. CK^+^ epithelial cells from the extrahepatic bile ducts (EHBD) or IHBD were counted at 40x magnification, followed by counts of BrdU^+^ cells. The percentage of BrdU-positive CK^+^ epithelial cells was determined for each animal. At least 500 cells per compartment (CK^+^ EHBD and CK ^+^ IHBD) were counted for each animal. Images shown of the EHBD are at 20x magnification, and at 40x magnification of the IHBD.

### Statistics

Data are presented as mean ± S.D. unless otherwise specified. One- or two-tailed Student’s t-test was used, with p<0.05 as specified in the figure legends. Numbers of samples per experimental group and numbers of experimental replicates are depicted in figures or in figure legends as appropriate.

### Study approval

The Cincinnati Children’s Hospital Medical Center IACUC approved the experimental protocols for the mouse experiments, protocol number: 2017–0007.

## Results

### Regional restriction of CD45^+^Lin^neg^ lymphocytes in the liver and EHBD

**Fig 1:**
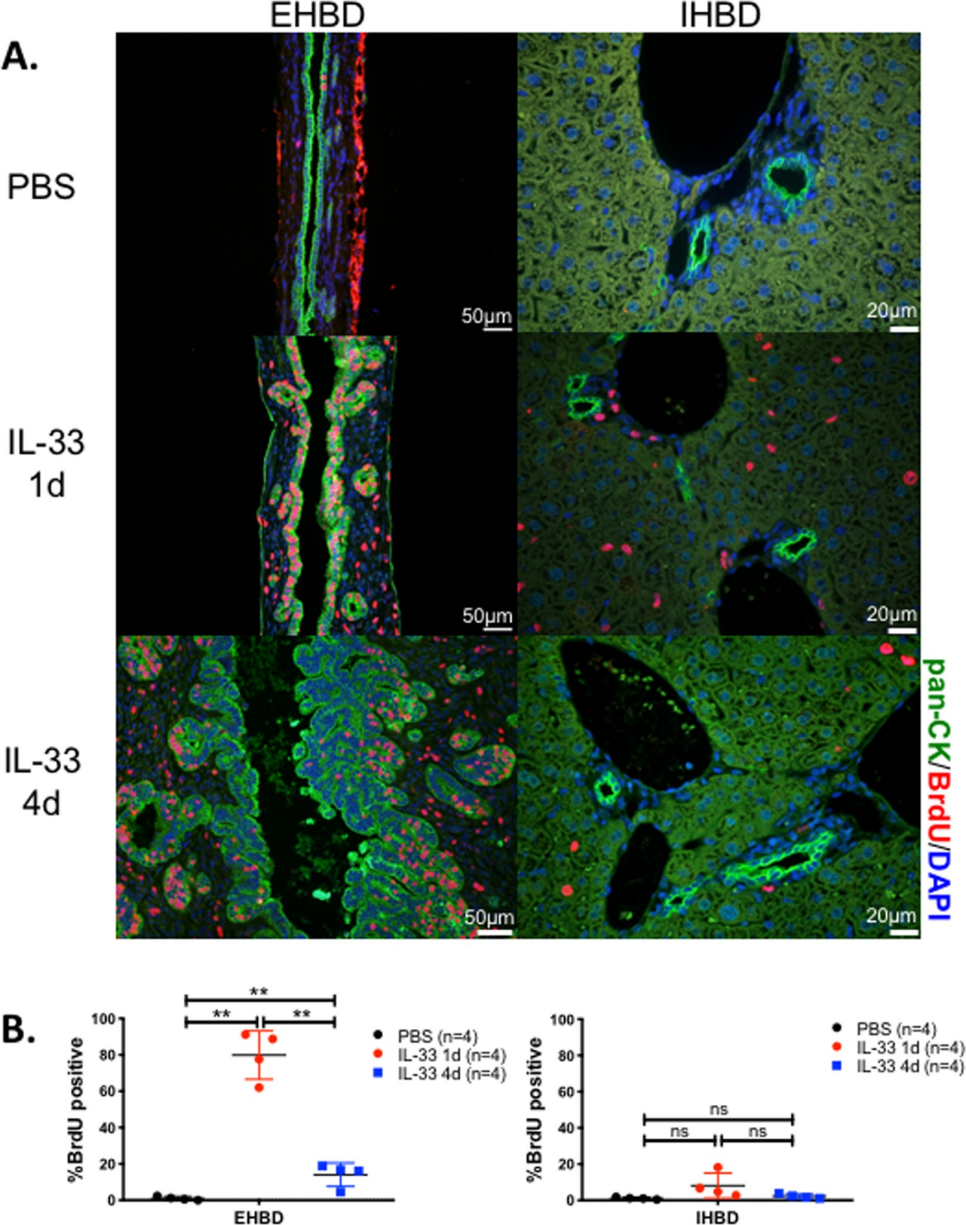
IL-33 induced cholangiocyte proliferation is restricted to the extrahepatic bile ducts. Balb/c mice (n = 2/group) were injected i.p. with PBS or IL-33 and tissues were harvested 24 hours or 4 days later. Two hours prior to harvest, BrdU was administered i.p. to mark rapidly proliferating cells. (A) Representative sections of EHBD and liver after immunofluorescence staining with anti-cytokeratin (CK;green) and anti-BrdU (red) antibodies with DAPI (blue) nuclear stain. (B) The percentage of BrdU^+^ cells among all CK^+^ cells. Data are combined from two independent experiments, n = 4 mice per group total. Mean +/- s.d. ** = p<0.005 by two-tailed Student’s t-test. ns = not significant.

To demonstrate the tissue-specific proliferative response of IL-33 prior to single-cell sequencing experiments, we first administered 1μg IL-33 or PBS daily i.p. to adult Balb/c mice and quantified BrdU^+^ cholangiocytes 24 hours later or after 4 daily doses of IL-33. One dose of IL-33 induced a significant increase in the number of BrdU^+^ cholangiocytes in the EHBD as compared to intrahepatic cholangiocytes (Fig 1). These data underscore different levels of cholangiocyte responsiveness to IL-33 between the intra- and extrahepatic domains of bile ducts.

Based on the role of IL-33 in the activation of ILCs, we next investigated how the functional maturation of ILCs is regulated by the tissue microenvironment in response to IL-33 by using single cell RNA sequencing of hepatic mononuclear cells. Mononuclear cells were isolated from livers of adult Balb/c mice treated with PBS (PBS-Liver, serving as baseline controls), from the livers 1 and 4 days after daily intraperitoneal injections of IL-33, and from EHBDs 4 days after daily intraperitoneal injections of IL-33. Because the number of CD45^+^ mononuclear cells existing in the PBS-treated EHBD was exceedingly low even after pooling more than 15 EHBDs, we did not include this control group. Mononuclear cells were stained with a cocktail of lineage-specific antibodies, which included markers for mature T cells (CD3, CD4, CD8), B cells (B220), myeloid cells (CD11b, CD11c, F4/80, Gr-1), mast cells (FcεRIα), NK cells (CD49b), and erythrocytes (TER-119). CD45^+^Lin^neg^ cells were sorted and subjected to single cell RNA sequencing using the C1 Fluidigm platform. The total cell yield with high-quality RNA for each experimental group varied between 39–82 cells as shown in S1 Table.

**Fig 2:**
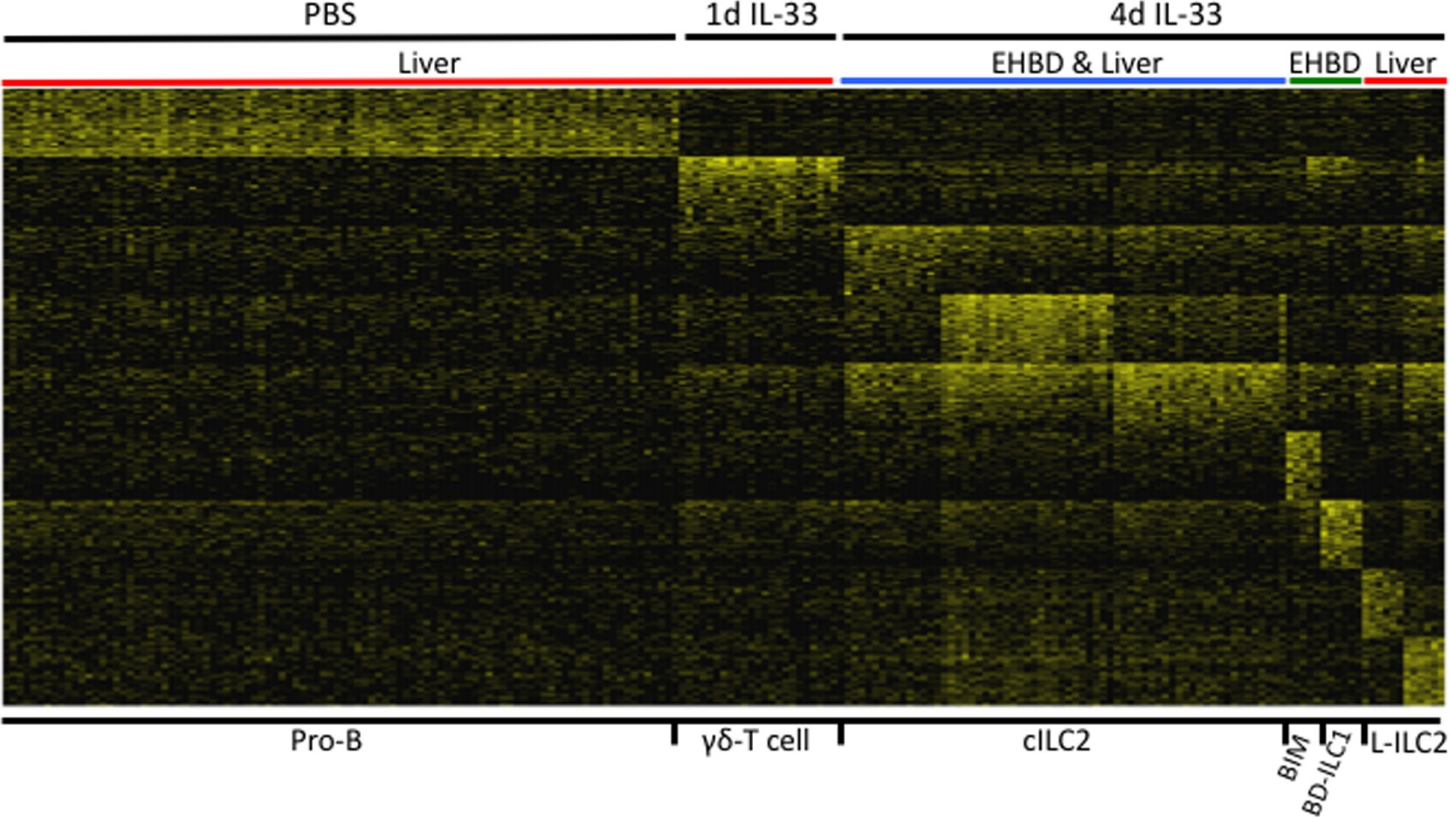
Single cell RNA sequencing identifies tissue-specific subtypes of innate lymphoid cells. Single cell RNA sequencing was performed on CD45^+^Lin^neg^ mononuclear cells isolated from the liver or EHBD from PBS- or IL-33-treated adult Balb/c mice as described in Methods. Cluster analysis using the top 200 genes expressed by each cell class identifies 6 major CD45^+^Lin^neg^ cell classes in the liver and/or EHBD in PBS- or IL-33 treated Balb/c mice. Gene expression is shown as a color gradient from yellow (high expression) to black (low expression) using log_2_(TPM+1) values.

To identify cell subtypes, we performed cluster analysis in the gene expression platform using a pipeline constructed in Python applying principal component analysis and an iterative code that included dimension reduction (feature selection), clustering, and signature gene identification[14]. This approach identified 17 different subpopulations of hepatobiliary CD45^+^Lin^neg^ mononuclear cells (S1 Fig). Because some CD45^+^Lin^neg^ subpopulations contained fewer than 5 cells, these cell classes were excluded from further analysis, allowing the identification of 6 major cell classes or class groups for further study as shown in Fig 2. We assigned a cellular identity to each major CD45^+^Lin^neg^ class based on the functional profiles of the top 200 genes expressed in each class; this approach revealed the most prevalent classes within each tissue (Table 1**)**. Using this approach, we named the 6 major classes as Pro-B cells, liver γδ-T cells (L-γδ), canonical ILC2 (c-ILC2), liver-specific ILC2 (L-ILC2), a bile duct specific class of ILC1 (BD-ILC1), and a previously undescribed class of biliary immature myeloid cells (BIM).

**Table 1:** Relative frequency of major cell classes among Lin^neg^CD45^+^ mononuclear cells isolated from liver or EHBD after PBS or IL-33 treatment.

	Pro-B (Liver)	L-γδ (Liver)	Canonical ILC2 (Liver & EHBD)	BIM (EHBD)	BD-ILC1 (EHBD)	Liver-ILC2 (Liver)	Total %^A^
**PBS Liver**	**76**	**7**	**0**	**0**	**0**	**0**	**83**
**Liver 1d IL-33**	**56**	**25**	**1**	**1**	**0**	**0**	**83**
**Liver 4d IL-33**	**18**	**3**	**53**	**0**	**0**	**16**	**90**
**EHBD 4d IL-33**	**3**	**3**	**56**	**11**	**16**	**0**	**89**
**Cell ID**	**Pro-B**	**γδ-T**	**ILC2**	**Myeloid**	**ILC1**	**ILC2**	
**Selected Highly**	***Ebf1***	***Nkg7***	***Hist1h4d***	***Gp49a***	***Cd28***	***Nudt21***	
**Expressed**	***Mef2c***	***Fcer1g***	***Arg1***	***Cd200r1***	***Lyst***	***Mien1***	
**Genes**	***CD79a***	***Ctsw***	***Ccr8***	***Lilrb4***	***Tagap***	***Ndufb6***	
	***Blnk***	***Ncr1***	***Cks1b***	***Cdc42ep3***	***fPnrc1***	***Polb***	
	***Ifi30***	***Klrd1***	***Top2a***	***Pear1***	***68204f20Rik***	***Myo1g***	
	***Syk***	***Xcl1***	***2810417H13***	***Tmem123***	***AK148373***	***Gadd45b***	
	***Cd55***	***Tyrobp***	***Smc2***	***AK202516***	***Satb1***	***Bbs9***	
	***H2-Eaps***	***Gzma***	***Rrm2***	***Mcpt8***	***Fam124b***	***Myadm***	
	***Swap70***	***Klrk1***	***Rqcd1***	***Cabin1***	***Klhl20***	***Arrdc1***	
	***Cd24a***	***Klrc1***	***Ccr2***	***Meaf6***	***Cebpb***	***Ddx47***	
	***Cd74***		***IL1r1***	***Ccr1***	***Ifngr1***	***Tnfsf4***	
			***Icos***		***CD93***	***Tnfsf9***	

^**A**^Total percentage of cells accounted for in each control or experimental group. The percentage of unaccounted cells are in the minor classes which are not included in this table.

Based on the data in Table 1, the Pro-B cell class expressed high levels of *IL-7R*, *Igh*, *CD55*, *CD79a*, and *CD74* transcripts and represented 76% of the CD45^+^Lin^neg^ liver mononuclear cells in the PBS-control group. Although cells of the Pro-B class were also found in other experimental conditions, they were present at much lower frequency. L-γδ T cells, identified by expression of *Ctsw*, *Gzma*, *Ncr1*, *TCRδ* and *TCRγ* transcripts, were present at the highest frequency in the liver after 1d IL-33 treatment, representing 25% of the cells in this treatment group. As expected, c-ILC2 cell classes were found both in the liver and EHBD in the 4d IL-33 treatment group. c-ILC2 expressed transcripts for transcription factors (*Rora*, *GATA3*), surface markers (*Arg1*, *CCR8*, *KLRG1*, *Icos*, *Il17Rb*, *Ccr2*) and type 2 cytokines (*Il5*, *Il13*) known to be associated with ILC2 [18,19]. A group of ILC2s uniquely expressing transcripts for *Ccr4*, *Tnfsf9*, *Il12rb1*, *IL10*, *NFil3*, *Idi1*, and *IL9r* were only found in the liver, but not EHBD, after 4d IL-33, thus named L-ILC2s to denote liver specificity. Two CD45^+^Lin^neg^ cell classes were restricted to the EHBD after IL-33 treatment (not present in the liver): BD-ILC1 and BIM. BD-ILC1 expressed transcripts consistent with ILC1 (*Cd28*, *Il12rb2*, *Thy1*, *Cd93*, *Tbx21*, *Ifngr1*, *Il2*, *Il10*), while BIM expressed genes consistent with immature myeloid cells (*Gp49a*, *Cd200r1*, *Lilrb4*, *Klf7*, *Ets2*, *Il6*, and *Il1b*).

**Fig 3:**
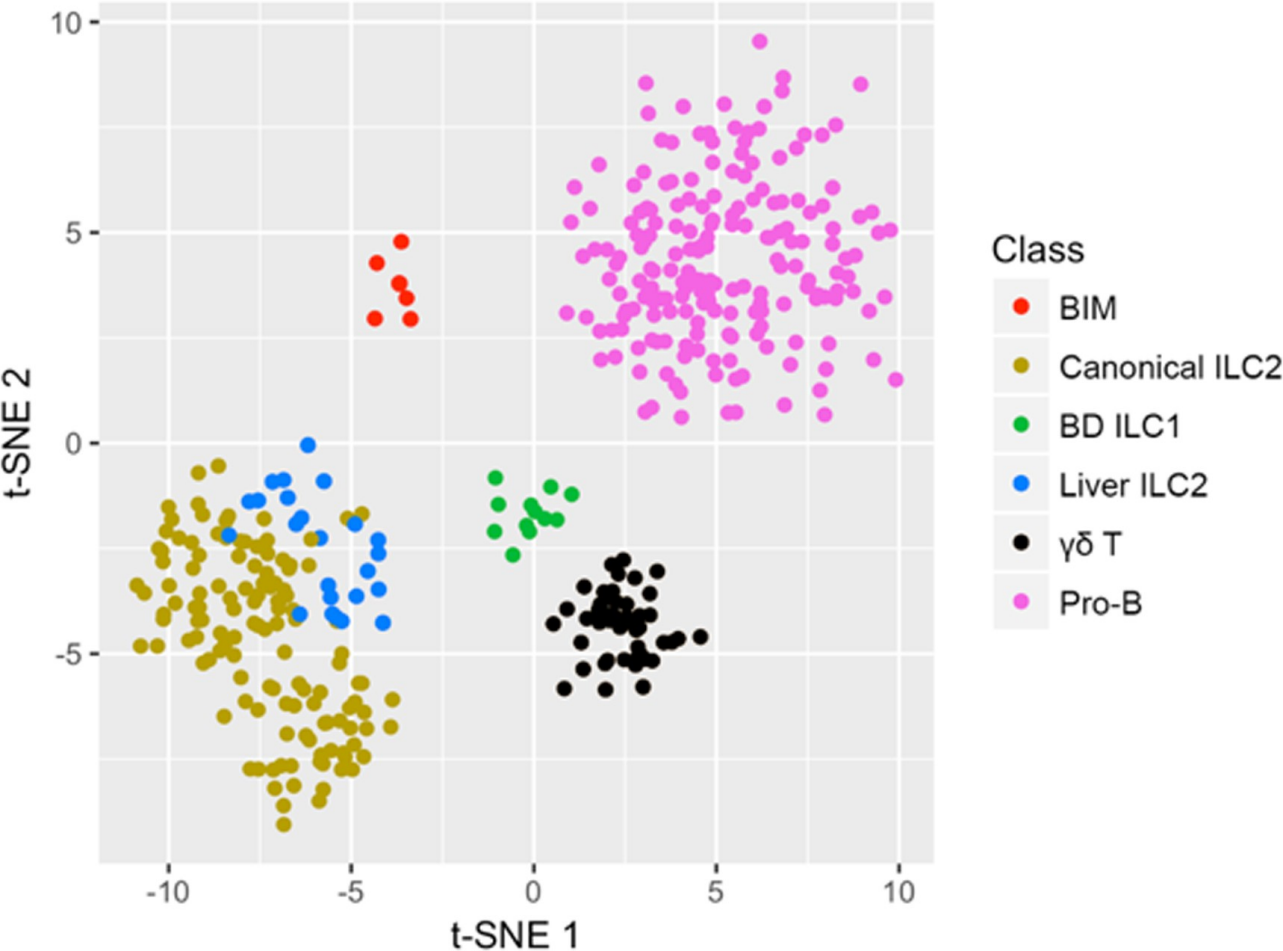
BIM are distinct from ILC2. Stochastic neighbor embedding plot (t-SNE) utilizing the top 200 genes in each major cell class shows the distinct position of BIM and its relatedness to other IL-33-induced hepatobiliary CD45^+^Lin^neg^ mononuclear cell classes.

Seeking insight into the level of distinction and potential relationships of c-ILC2 and BIM and other Lin^neg^ mononuclear cells, we utilized stochastic neighbor embedding (t-SNE) analysis of the gene sets of the 6 major cell classes described above [19]. We found that c-ILC2 and BIM remain as separate groups (Fig 3).

**Fig 4:**
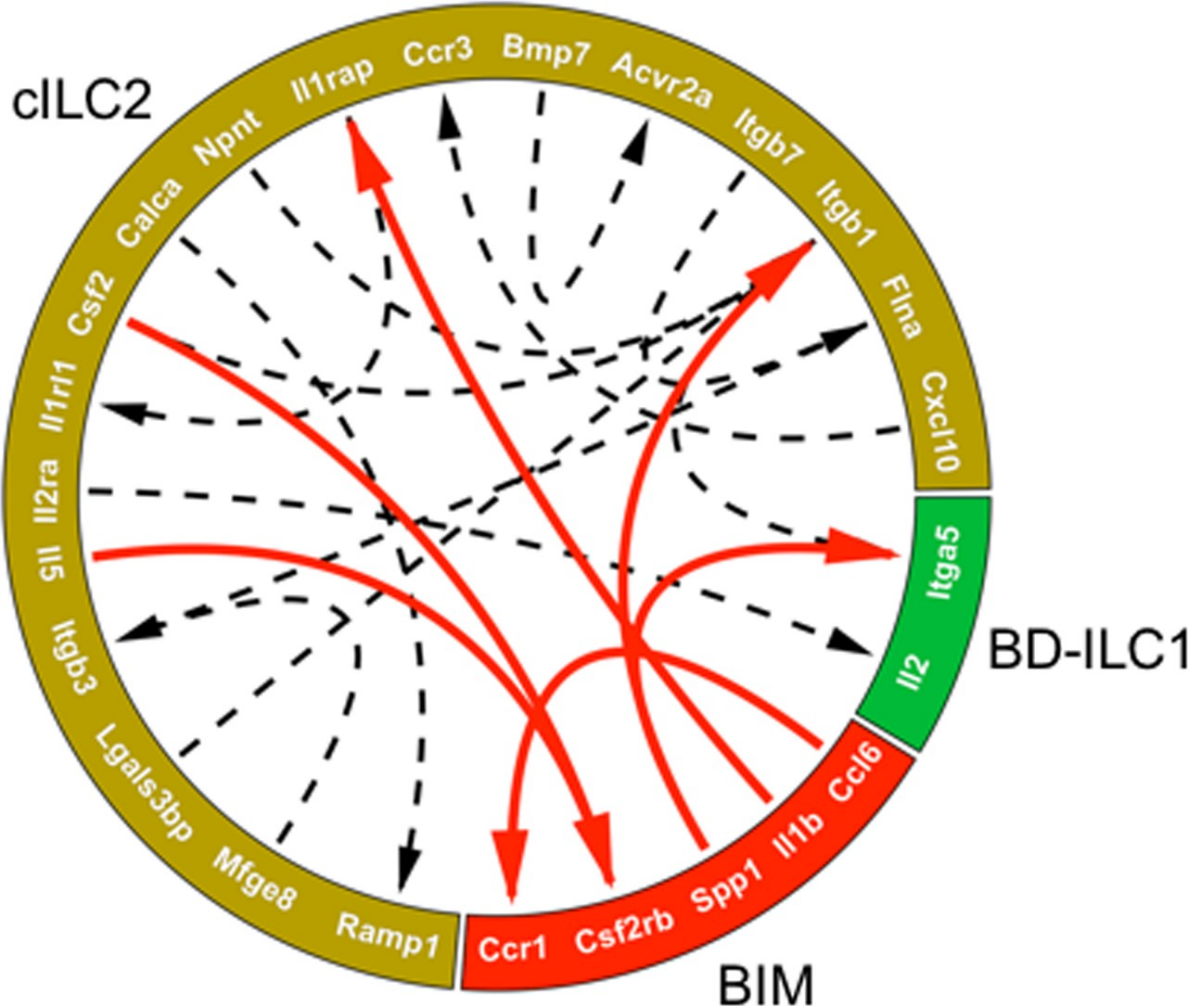
BIM are predicted to interact with ILC2. Predicted receptor-ligand interactions between BIM and either BD-ILC1 or cILC2 isolated from the EHBD (red arrows). Dashed lines represent predicted interactions between cILC2 and BD-ILC1 cell classes in the EHBD.

Because the proliferative response of cholangiocytes to IL-33 is predominantly restricted to EHBDs, we focused next on identifying potential cell-cell interactions among the three cell classes present in EHBD: c-ILC2, BD-ILC1, and BIM. We used the Circlize program in the R platform to probe the gene list of the 3 major cell classes in the bile duct for potential receptor-ligand interactions (Fig 4). The approach predicted a total of 15 receptor-ligand interactions (dashed lines), 6 of which occurred with BIM as a node, of which 66% were with cells of the cILC2 classes (red lines). These data suggest that BIM could potentially interact directly with ILC2 at the site of IL-33 driven cholangiocyte proliferation in the EHBD.

### Validation of gene expression signatures of BIM and BD-ILC1 at the tissue level

**Fig 5:**
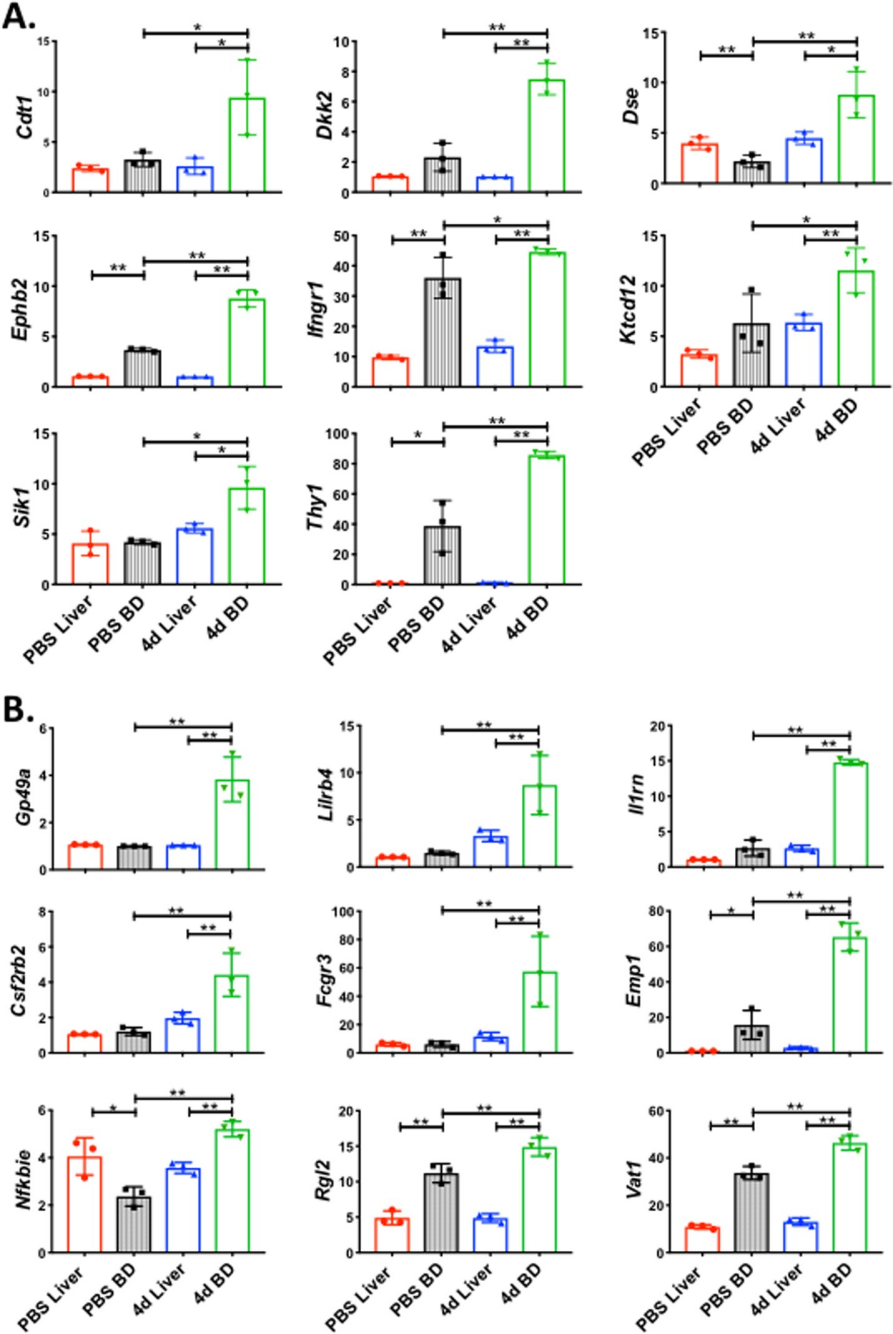
The signatures of BIM cells and BD-ILC1 cells are reproducible at the tissue level and restricted to the extrahepatic bile duct. RNA sequencing was performed on liver or bile duct tissue isolated from mice treated with either PBS or IL-33 for 4 days. BIM-specific genes (**A**) and BD-ILC1 specific genes (**B**) are selectively upregulated in the EHBD after IL-33 treatment (4d BD) relative to IL-33 treated liver (4d Liver) and PBS control tissues. N = 3 samples per group. Data are presented as mean ± s.d., * = p<0.05, ** = p<0.005 by one-tailed Student’s t-test. The gene expression signatures of the remaining 4 major cell classes were not reproduced at the tissue level.

To provide first-order validation of the cell classes in EHBDs (cILC2, BIM, and BD-ILC1), we first manually curated the gene expression sets for c-ILC2, BIM, and BD-ILC1, selecting only those genes which were expressed at a threshold log_2_(TPM+1) value >2 and not expressed in any other cell class, focusing our efforts on genes encoding cell-surface and secreted proteins. Then, we performed RNAseq using liver and EHBD isolated from Balb/c mice after administration of PBS or daily doses of IL-33 for 4 days (4d IL-33). Using Genespring software to analyze the data from RNAseq, we detected the expression of statistically significant gene expression for 9 of the 38 BIM-specific genes and 8 of the 72 BD-ILC1-specific genes at the tissue level, while the gene groups for the other classes had limited or no detectable expression in the EHBD (Fig 5, Table 2). Therefore, the single-cell signatures for BIM and BD-ILC1 are reproducible at the tissue level, with increased expression in the EHBD after IL-33 treatment.

**Table 2:** Validation of the BIM and BD-ILC1 gene signature at the tissue level.

Cell Class	# genes	# genes with signal^A^	# genes with significant expression
**Pro-B**	18	17	1
**L-γδ**	20	15	1
**c-ILC2**	20	19	0
**BIM**	38	35	9
**BD-ILC1**	72	52	8
**L-ILC2**	123	110	2

^A^Signal is defined as the presence of detectable tissue mRNA expression by RNAseq.

### Expression of Gp49 on the surface of BIM cells

After successfully validating the BIM and ILC1 subclasses at the RNA level in the IL-33 treated EHBD, we next sought to identify these cell classes by flow cytometry using cell-class associated proteins (Table 1). Mononuclear cells were isolated from PBS- or IL-33 treated Balb/c mice, and analyzed by flow cytometry for the expression of cell-class associated receptors on the cell surface. No detectable expression of the BD-ILC1-class associated markers CD28, CD93, or the type 1 interferon gamma receptor was found on CD45^+^Lin^neg^ mononuclear cells in the liver or EHBD in either control or 4d IL-33 treatment conditions (S2 Fig).

**Fig 6:**
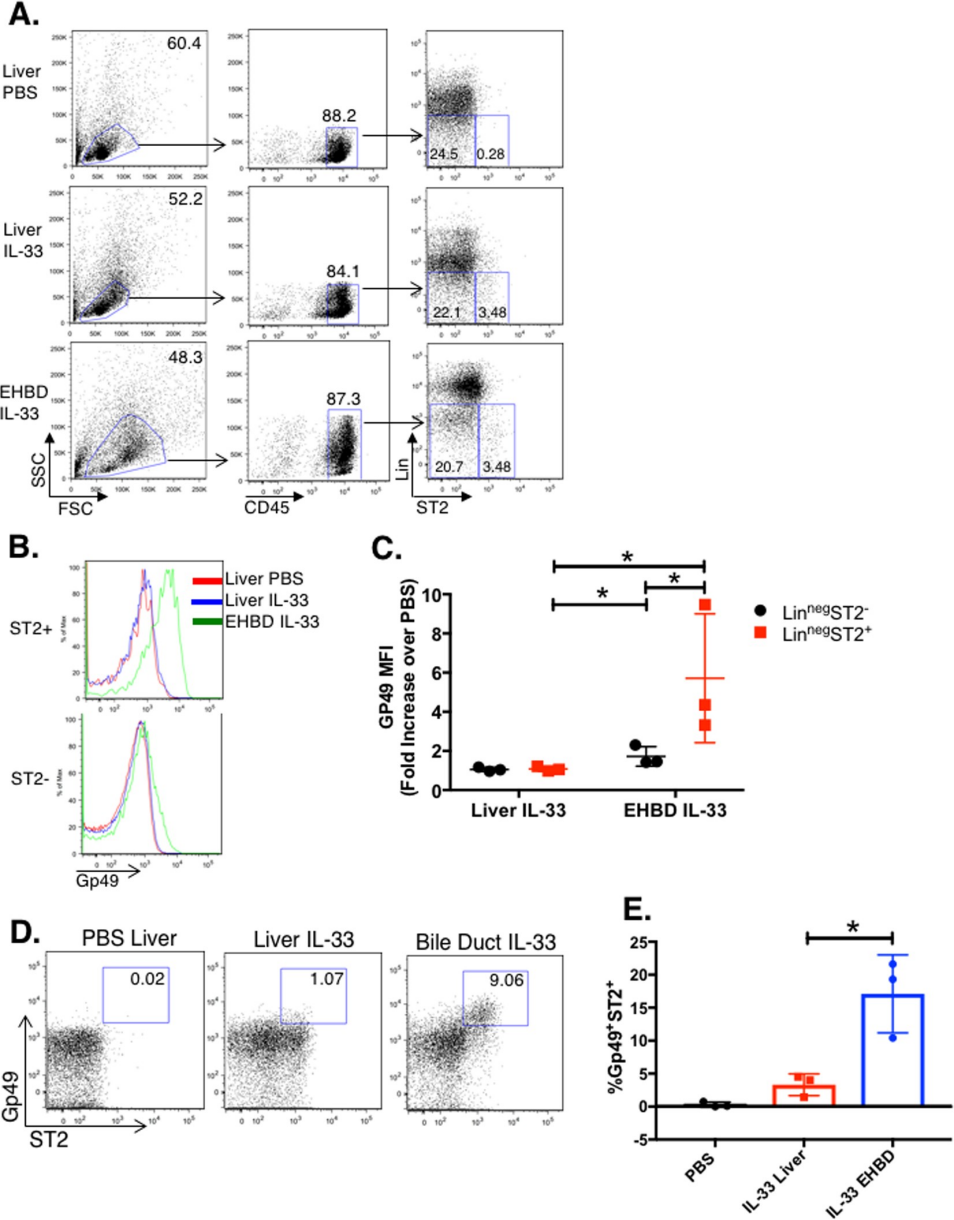
Validation of the BIM cell class by flow cytometry. Mice were treated with either PBS or IL-33 for 4 days, after which mononuclear cells were isolated from liver and EHBD tissue as described in Methods, stained with florescent antibodies, and analyzed by flow cytometry. Gating strategy (**A**) to identify CD45^+^Lin^neg^St2^+^ vs. St2^-^ mononuclear cells in liver and EHBD after PBS- or IL-33 treatment (representative of 3 independent experiments). **(B)** shows relative expression of BIM cell-class specific marker, Gp49, in ST2^+^ vs. ST2^-^CD45^+^Lin^neg^ mononuclear cells isolated from PBS-treated liver (red histogram) or IL-33-treated liver (blue histogram) or EHBD (green histogram); (representative of 3 independent experiments). (**C**) shows Gp49 MFI, expressed as fold-increase over PBS (combined from 3 independent experiments), * = p<0.05 by one-tailed Student’s t-test. Representative dots plots (**D**) and quantification from 3 independent experiments (**E**) of flow cytometric assays show that BIM are induced specifically in the extrahepatic bile duct but not in liver after IL-33 treatment. Mean ± s.d., 3 replicates, * = p<0.05 by 2-tailed Student’s t-test.

We next sought to identify the BIM class using the class-associated markers, CCR1 and Gp49, by flow cytometry. Mononuclear cells were isolated from livers and EHBDs of Balb/c mice after 4 daily doses of IL-33 or PBS, and subjected to flow cytometric analysis. Liver and EHBD mononuclear cells were gated as shown in Fig 6A. CCR1 was not identified on CD45^+^Lin^neg^ mononuclear cells after IL-33 treatment (S3 Fig). Using the gating strategy in Fig 6A, Gp49 expression was compared between CD45^+^Lin^neg^ST2^+^ and ST2^-^ populations. Quantification of Gp49 MFI showed the highest expression in CD45^+^Lin^neg^ST2^+^ mononuclear cells from EHBD relative to PBS control and liver with or without IL-33 treatment, and lower but detectable levels on CD45^+^Lin^neg^ST2^-^ mononuclear cells (Fig 6B and 6C). When gated on total Lin^neg^ cells, the Gp49^+^ST2^+^ subpopulation made up ~15% of the CD45^+^Lin^neg^ cells of EHBDs (Fig 6D and 6E**).**

**Fig 7:**
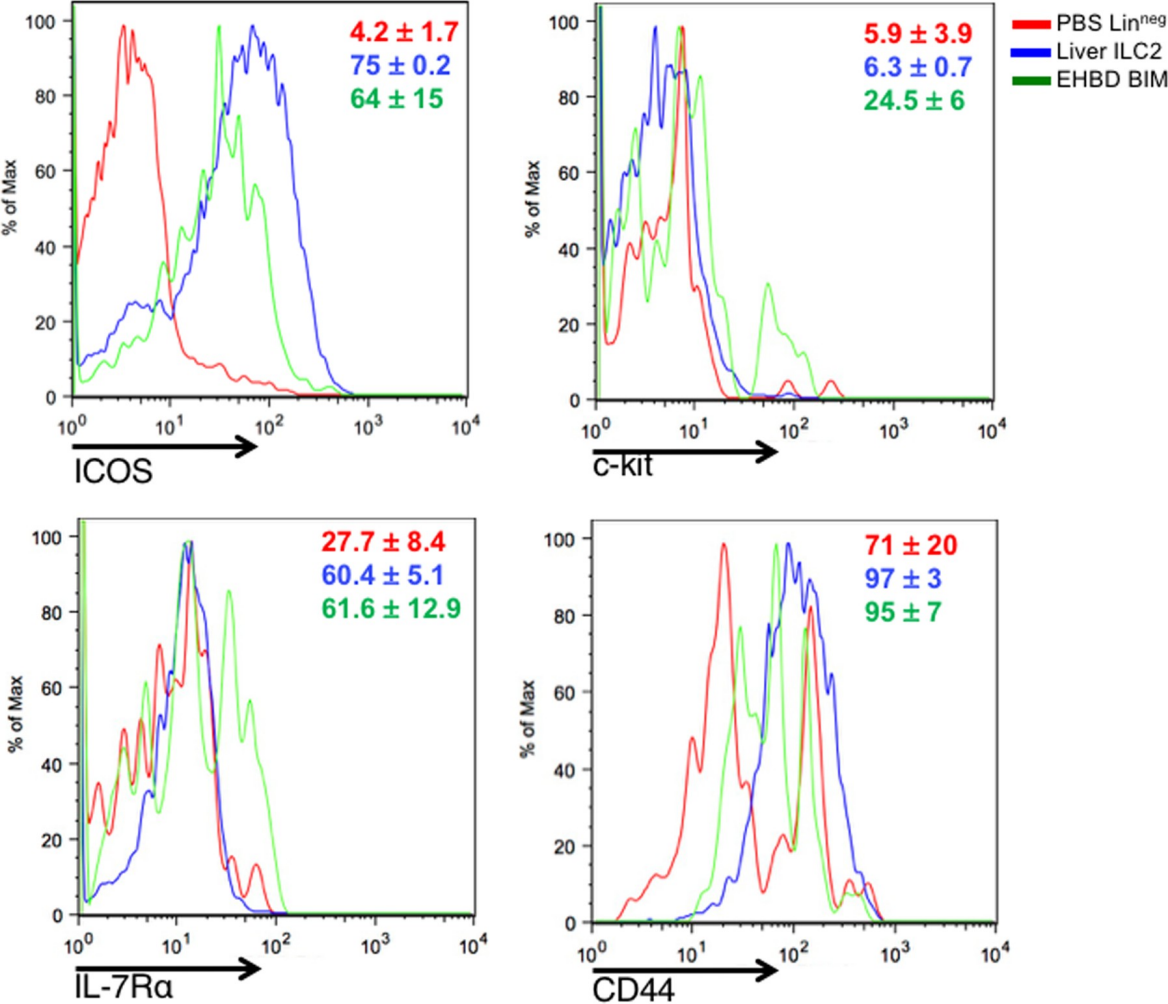
Biliary immature myeloid cells differentially express c-kit. Liver and EHBD mononuclear cells from IL-33 treated mice were analyzed by flow cytometry. Cells were initially gated on CD45^+^Lin^neg^ lymphocytes and expression of c-kit, IL-7Ra, and CD44 was compared between biliary immature myeloid cells (Lin^neg^Gp49^+^ST2^+^; green histogram), hepatic ILC2 (Lin^neg^ST2^+^; blue histogram), and Lin^neg^ lymphocytes isolated from PBS-treated liver (red histogram). Relative cell frequencies are shown from 2–3 independent experiments, mean +/- s.d. in inset.

To examine how this subpopulation is phenotypically related to ILC2s, we determine the expression of the canonical activation markers ICOS, CD44, c-kit, and IL-7Rα in Gp49^+^ cells [7]. Gp49^+^ST2^+^ BIM cells expressed similarly high levels of ICOS, CD44, and IL-7Rα compared to c-ILC2, but differed from ILC2s by the increased expression of c-kit (Fig 7). Interestingly, this coincides with a recent report describing differential c-kit expression in human ILC2, with the c-kit^hi^ population demonstrating increased cytokine secretion in response to stimulation with the c-kit ligand, SCF [20]. Combined, these data suggested that BIM are CD45^+^Lin^neg^ ST2^+^Gp49^+^ cells enriched in EHBDs after IL-33 administration and display an activation profile of ICOS, CD44, and IL-7Rα expression.

## Discussion

In this report, we used single cell RNA sequencing to characterize the heterogeneity of the hepatobiliary ILC compartment in control and IL-33 treated mice. Using this powerful technique, we identified a new subset of innate EHBD-specific BIM cells which express high levels of Gp49 and are predicted to interact with c-ILC2.

IL-33, by inducing IL-13 production in ILC2s, has been shown to have seemingly disparate roles in promoting tissue repair and cholangiocyte proliferation in EHBDs while also exacerbating liver fibrosis[7,10,11]. While the proliferative response of cholangiocytes to intraperitoneal administration of IL-33 was restricted to the EHBD [7], the constitutive overexpression of IL-33 in the liver was shown recently to also induce proliferation of intrahepatic cholangiocytes [11,21]. Here, we took a step further by investigating the potential role the microenvironment may exert on the functional maturation of ILC2s in response to IL-33. IL-33 exposure triggered the emergence of cell subtypes that can be identified by the differential enrichment of gene transcripts. Among them, ILC2s are the most abundant and populate the liver and EHBDs. In addition, cell subgroups preferentially populated the liver (not EHBDs) such as the ILC2s expressing *Ccr4*, *Tnfsf9*, *Il12rb1*, *IL10*, *NFil3*, *Idi1*, and *IL9r*, while others populated primarily EHBDs such as the subgroup with a transcriptional signature of *Cd28*, *Il12rb2*, *Thy1*, *Cd93*, *Tbx21*, *Ifngr1*, *Il2*, *Il10* (more typical of ILC1), and the subgroup with transcripts more typical of immature myeloid cells (*Gp49a*, *Cd200r1*, *Lilrb4*, *Klf7*, *Ets2*, *Il6*, and *Il1b*). Bioinformatics analysis of transcripts encoding receptors and ligands in these cell groups suggested the existence of a rich interactive relationship among cell types, but we have not yet formally investigated the full extent of this possibility experimentally.

The anatomic restriction of individual cell subgroups forms a biological model that allows for the investigation of how molecular signals may induce diverse cellular responses linked to proliferation or fibrogenesis. Exploring this model, we performed initial studies in immature myeloid cells expressing Gp49. Like ILC2s, these cells express the canonical activation markers ICOS, CD44, c-kit, and IL-7Rα, but differ by the increased expression of c-kit. In EHBD, the presence of Gp49 and the transcriptional profile described herein identify a cell subgroup restricted to biliary tissue. These findings support a setting in which Gp49-expressing cells participate in a paracrine inter-cellular crosstalk that promotes proliferation of the biliary epithelium, but future studies are required to formally address this possibility.

In summary, we show the emergence of tissue-specific subpopulations of innate lymphoid cells linked to the multi-functional cytokine IL-33, with a mRNA profile predicting the expression of receptors and ligands that may indicate the existence of an inter-cellular network that cooperates to regulate cholangiocyte proliferation. Future studies are needed to define the function(s) of individual cell subpopulations, and to investigate whether Gp49-expressing cells produce IL-13 or other ligands that mediate epithelial expansion and promote repair following tissue injury.

## Supporting information

S1 Fig Single cell RNA sequencing reveals 17 unique cell classes of CD45^+^Lin^neg^ mononuclear cells in the liver and extrahepatic bile duct. Cluster analysis of the top 200 genes identifies 17 total CD45^+^Lin^neg^ cell classes in the liver and/or EHBD in PBS- or IL-33 treated Balb/c mice. Gene expression is shown as a color gradient from yellow (high expression) to black (low expression) using log_2_(TPM+1) values.
(TIF)

S2 Fig BD-ILC1 associated proteins are not detected in hepatobiliary Lin^neg^ mononuclear cells. Mice were treated with either PBS or IL-33 for 4 days, after which mononuclear cells were isolated from liver and EHBD as described in Methods, stained with fluorescent antibodies, and analyzed by flow cytometry. Cells were gated as shown in Fig 6 to identify CD45^+^Lin^neg^ST2^+^ vs. ST2^-^ mononuclear cells in liver and EHBD after PBS- or IL-33 treatment. Relative expression of the BD-ILC1 cell class associated markers CD28 and CD93 in ST2^+^ vs. ST2^-^ CD45^+^Lin^neg^ mononuclear cells isolated from PBS-treated liver (red histogram) and IL-33 treated liver (blue histogram) and EHBD (green histogram) is shown; histograms are representative of 3 independent experiments.
(TIF)

S3 Fig CCR1, a BIM cell class associated protein, is not detected in hepatobiliary Lin^neg^ mononuclear cells. Mice were treated with either PBS or IL-33 for 4 days, after which mononuclear cells were isolated from liver as described in Methods, stained with fluorescent antibodies, and analyzed by flow cytometry. Cells were gated as shown in Fig 7 to identify CD45^+^Lin^neg^ST2^+^ vs. ST2^-^ mononuclear cells in liver after PBS- or IL-33 treatment. Relative expression of the BIM cell associated marker CCR1 in ST2^+^ vs. ST2^-^ CD45^+^Lin^neg^ mononuclear cells isolated from PBS-treated liver (red histogram), IL-33 treated liver (blue histogram) EHBD (green histogram) is shown; histograms are representative of 3 independent experiments.
(TIF)

S1 Table Total cell yield of liver and EHBD mononuclear cells for single-cell RNA sequencing. (DOCX)

S1 File PBS and IL33 treated whole Liver and BD gene expression matrix TPM value. (ZIP)

S2 File PBS and IL33 treated single cell gene expression matrix TPM value. (ZIP)
