# Supplementary figures and images for: Single cell RNA sequencing reveals regional heterogeneity of hepatobiliary innate lymphoid cells in a tissue-enriched fashion
Source: PLoS One. 2019 Apr 25;14(4):e0215481. doi: 10.1371/journal.pone.0215481 (PMC6483339; doi:10.1371/journal.pone.0215481)

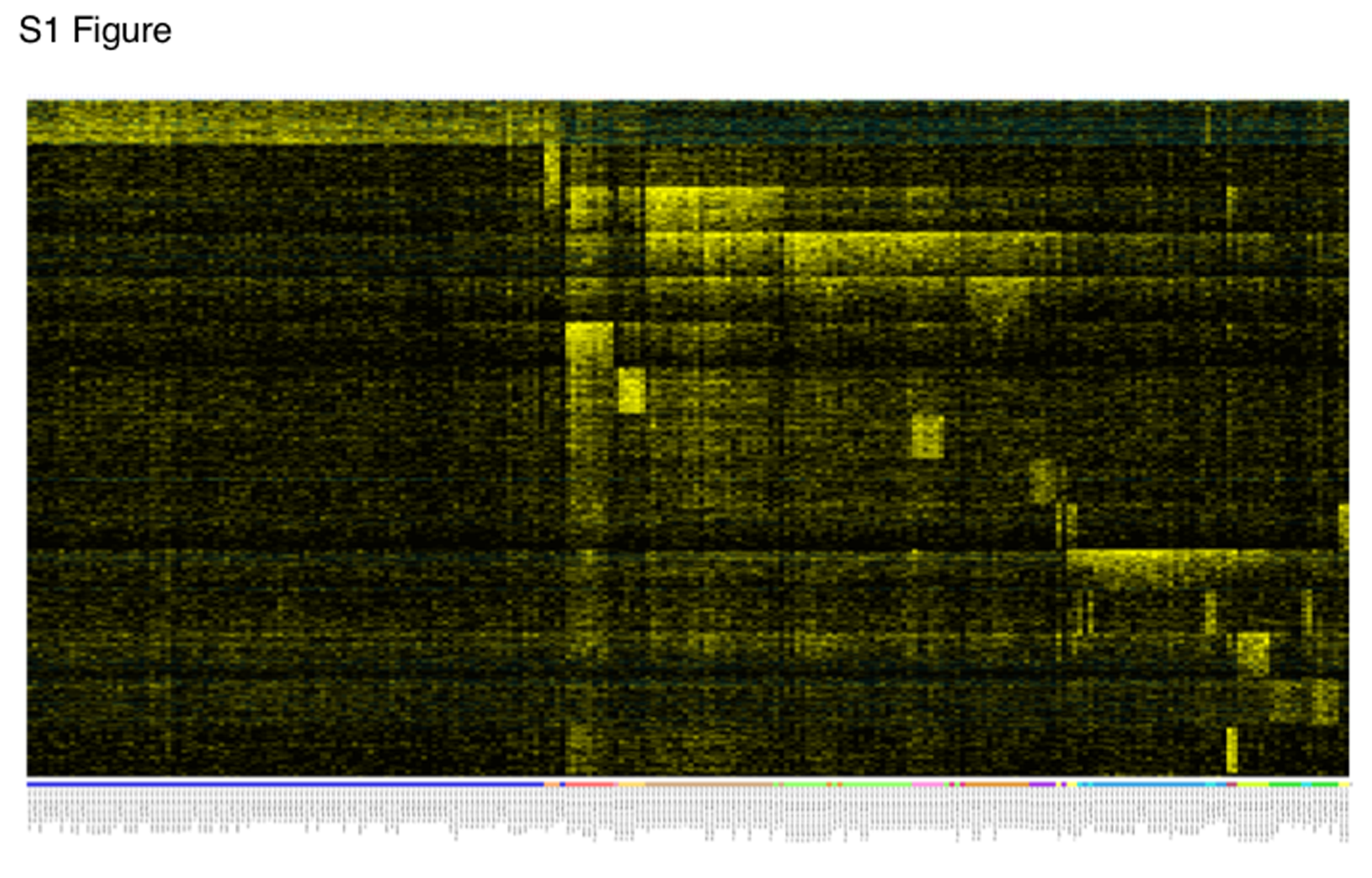

Supplement: S1 Fig — Cluster analysis of the top 200 genes identifies 17 total CD45+Linneg cell classes in the liver and/or EHBD in PBS- or IL-33 treated Balb/c mice. Gene expression is shown as a color gradient from yellow (high expression) to black (low expression) using log2(TPM+1) values. (TIF) [file pone.0215481.s001.tif]

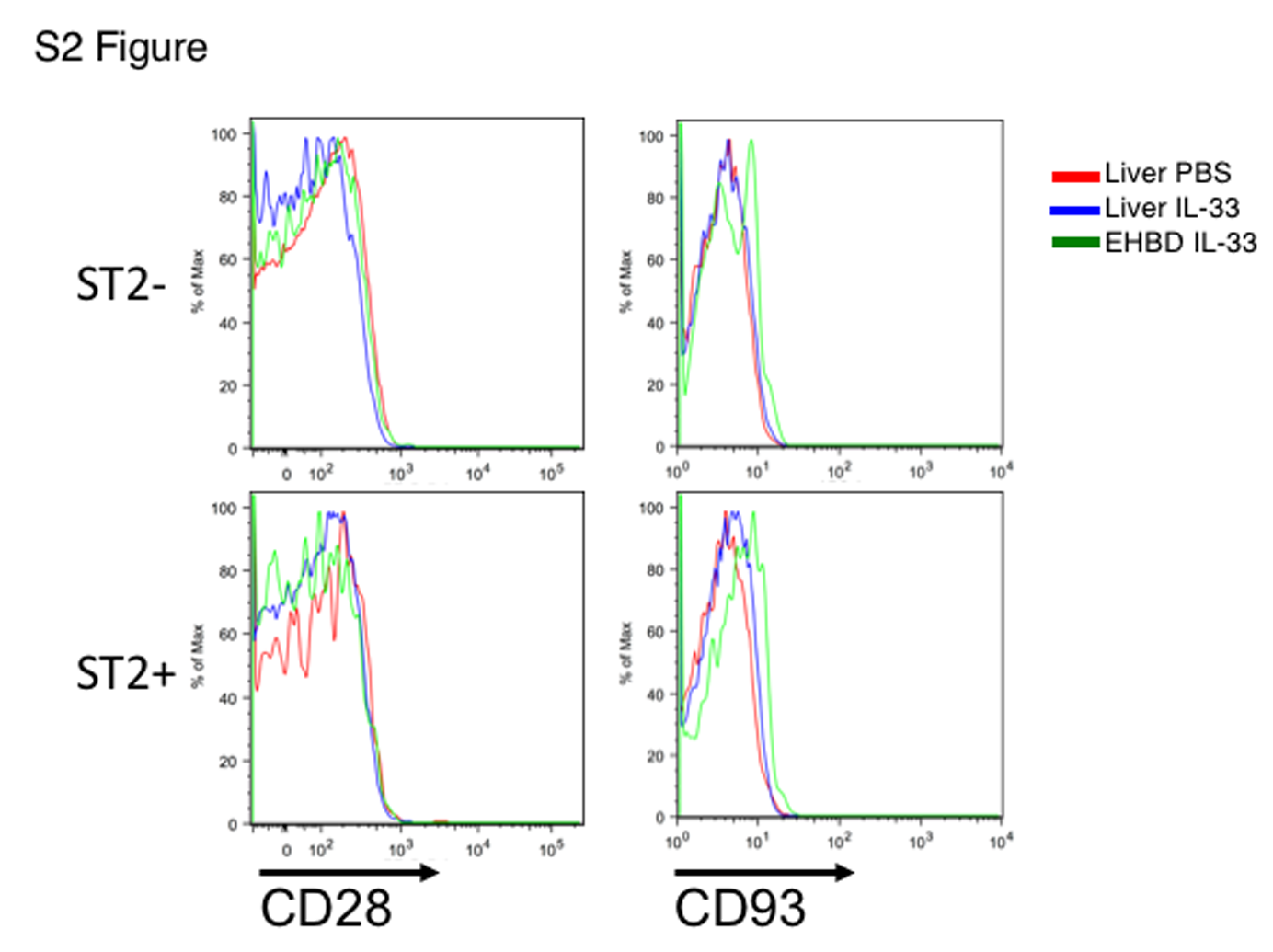

Supplement: S2 Fig — Mice were treated with either PBS or IL-33 for 4 days, after which mononuclear cells were isolated from liver and EHBD as described in Methods, stained with fluorescent antibodies, and analyzed by flow cytometry. Cells were gated as shown in Fig 6 to identify CD45+LinnegST2+ vs. ST2- mononuclear cells in liver and EHBD after PBS- or IL-33 treatment. Relative expression of the BD-ILC1 cell class associated markers CD28 and CD93 in ST2+ vs. ST2- CD45+Linneg mononuclear cells isolated from PBS-treated liver (red histogram) and IL-33 treated liver (blue histogram) and EHBD (green histogram) is shown; histograms are representative of 3 independent experiments. (TIF) [file pone.0215481.s002.tif]

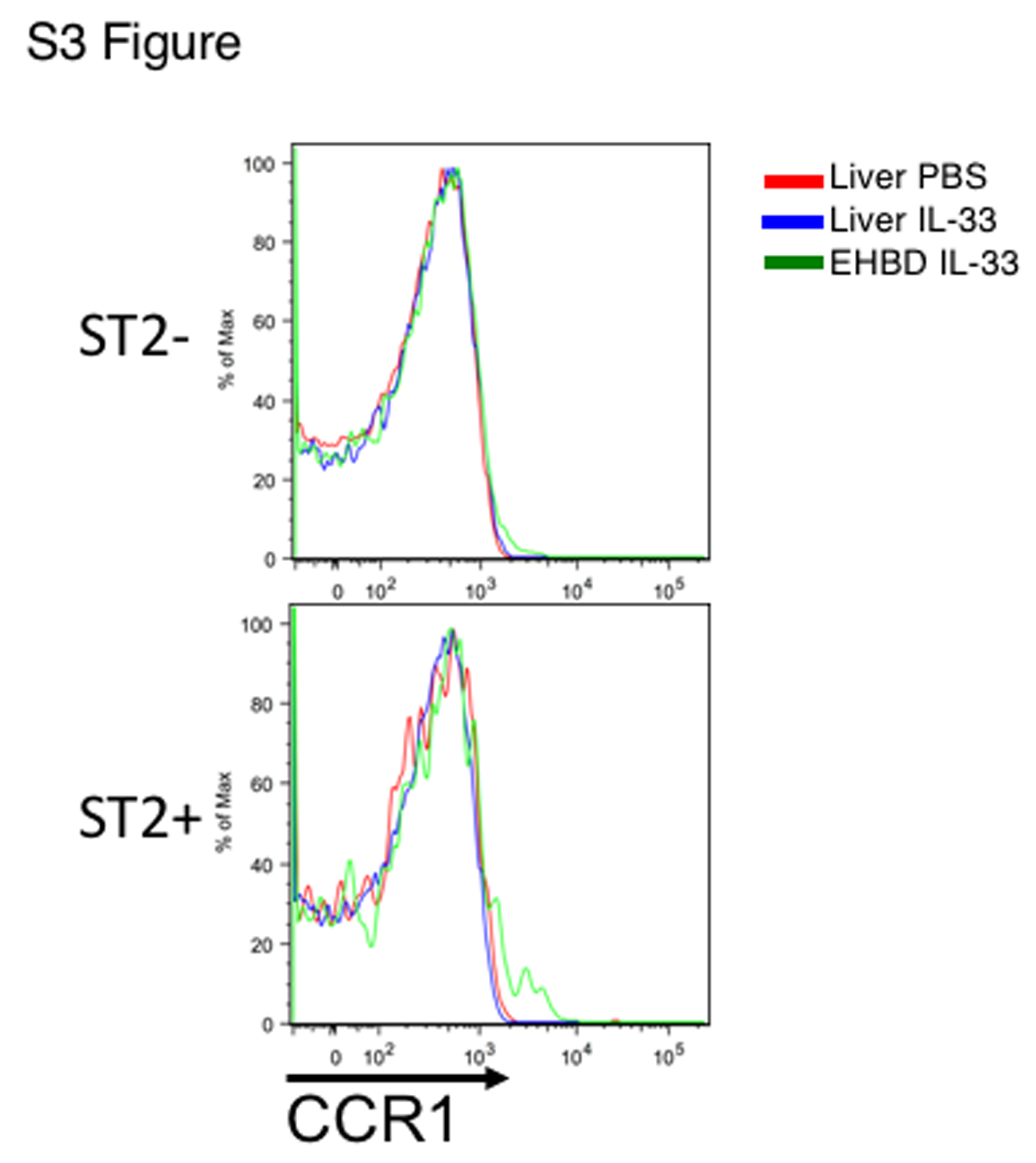

Supplement: S3 Fig — Mice were treated with either PBS or IL-33 for 4 days, after which mononuclear cells were isolated from liver as described in Methods, stained with fluorescent antibodies, and analyzed by flow cytometry. Cells were gated as shown in Fig 7 to identify CD45+LinnegST2+ vs. ST2- mononuclear cells in liver after PBS- or IL-33 treatment. Relative expression of the BIM cell associated marker CCR1 in ST2+ vs. ST2- CD45+Linneg mononuclear cells isolated from PBS-treated liver (red histogram), IL-33 treated liver (blue histogram) EHBD (green histogram) is shown; histograms are representative of 3 independent experiments. (TIF) [file pone.0215481.s003.tif]
