# Supplementary material for: Single cell RNA sequencing reveals regional heterogeneity of hepatobiliary innate lymphoid cells in a tissue-enriched fashion
Source: PLoS One. 2019 Apr 25;14(4):e0215481. doi: 10.1371/journal.pone.0215481 (PMC6483339; doi:10.1371/journal.pone.0215481)
Supplement: S1 Table — (DOCX) [file pone.0215481.s004.docx]

**Table S1: Total cell yield of liver and EHBD mononuclear cells for single-cell RNA sequencing.**

|  | **Total cells by microscopy** | **Total live single cells by microscopy** | **Cells with high quality/quantity cDNA** | **Cell # used in heatmap** |
| --- | --- | --- | --- | --- |
| **PBS Liver** | 92 | 85 | 82 | 80 |
| **Liver 1d IL-33** | 79 | 62 | 59 | 59 |
| **Liver 4d IL-33** | 80 | 68 | 68 | 68 |
| **EHBD 4d IL-33** | 51 | 43 | 39 | 38 |
